# Homoharringtonine inhibited breast cancer cells growth via miR-18a-3p/AKT/mTOR signaling pathway

**DOI:** 10.7150/ijbs.44907

**Published:** 2021-03-02

**Authors:** Li-bin Wang, Dan-ni Wang, Li-gang Wu, Jia Cao, Jin-hai Tian, Rong Liu, Rong Ma, Jing-jing Yu, Jia Wang, Qi Huang, Wen-yong Xiong, Xu Zhang

**Affiliations:** 1 The General Hospital of Ningxia Medical University, Biochip Research Center, Yinchuan, 750001, China.; 2 Gansu Provincial Hospital, Clinical Laboratory Center, Lanzhou, 730000, China.; 3 Key Laboratory of Medicinal Chemistry for Natural Resource, Yunnan University, Kunming, 650091, China.

**Keywords:** HHT, breast cancer, miR-18a-3p, AKT-mTOR signal pathway

## Abstract

Homoharringtonine (HHT), a natural alkaloid derived from the *cephalotaxus*, exhibited its anti-cancer effects in hematological malignancies clinically. However, its pesticide effects and mechanisms in treating solid tumors remain unclear. In this study, we found that HHT was capable of inhibiting tumor growth after 5-days treatment of breast cancer cells, MCF-7,* in vivo*. Furthemore, HHT also significantly inhibited the cancer cell growth and induced cell apoptosis *in vitro*. miRNA sequencing proved miR-18a-3p was noticeably downregulated in the cells after HHT treatment. Moreover, downregulating miR-18a-3p increased HHT-induced cell apoptosis; our data supported that HHT suppressed miR-18a-3p expression and inhibited tumorigenesis might via AKT-mTOR signaling pathway. In conclusion: our study proved that HHT suppressed breast cancer cell growth and promoted apoptosis mediated by regulating of the miR-18a-3p-AKT-mTOR signaling pathway, HHT may be a promising antitumor agent in breast cancer treatment.

## Introduction

Breast cancer is one of the leading causes of cancer-related deaths in women. The epidemiological survey showed that the incidence of breast cancer in the world is increasing year by year 1. Most patients with this type of cancer are diagnosed at an advanced stage of the disease, and the prognosis of these patients is still in poverty 2. At present, adjuvant chemotherapy and radiotherapy after surgical resection are the most commonly treatment strategies for breast cancer. However, multidrug resistance and toxicity of chemotherapy medicine have often influenced the outcome of the treatment 3, 4. Although a variety of chemotherapeutic medicine and target therapy are currently used in clinical, the median survival of patients with metastatic breast cancer was not improved significantly 5, 6. Therefore, to investigating new medicine for breast cancer treatment became urgent.

Homoharringtonine (HHT), an alkaloid which extracted from *cephalotaxus*, has been approved by the USA FDA for treatment of patients with hematological malignancies. HHT exerts its anti-tumor effects by fixing to ribosomes, disabling elongation of the nascent peptide chain and further protein synthesis 7, 8. It has proved that HHT could induce leukemia cell apoptosis by inhibiting expression of the apoptosis-related proteins 9. Several studies indicated that HHT also has anti-cancer effects on solid tumors. Wang et.al reported that HHT induced apoptosis and inhibited STAT3 via IL-6-JAK1-STAT3 signaling pathway in lung cancer cells 10. HHT also performed its anti-cancer activity by altering the characteristics of immune cells in the Kras mutation non-small cell cancer cells 11. A recent study reported that HHT suppressed triple negative breast cancer growth through reducing the expression of apoptosis proteins 12. The results indicated that HHT could be a potential therapeutic medicine for breast cancer. However, the anti-cancer effects of HHT on breast cancer and the underlying mechanisms are not clear yet.In this study, we investigated the effects of HHT on human breast cancer cells and studied the underlying molecular mechanism. We provided evidences that HHT inhibited breast cancer cell growth and induced apoptosis by regulating the miR-18a-3p-AKT-mTOR signaling pathway, supporting the HHT could be applied in breast cancer treatment in future.

## Materials and Methods

### Clinical samples collection

The study obtained approval from the Ethics Committee of General Hospital of Ningxia Medical University, which was conducted in accordance with the Declaration of Helsinki. Six paired breast cancer tissues and adjacent non-cancerous tissue samples were collected from the department of oncology.

### Cell culture and transfection

Homoharringtonine (HHT) was purchased from Hangzhou Minsheng Pharmaceutical Co, Ltd (Hangzhou, China). Human breast cancer cell lines MDA-MB-231, MCF-7, T47D, HCC1937 and MCF-10A were purchased from the American Type Culture Collection (ATCC, Mannasas,VA,USA). The cells were routinely maintained in Dulbecco's modified Eagle's medium (DMEM, HyClone, Logan, UT, USA), including 10% fetal bovine serum (FBS, Invitrogen, Carlsbad, CA, USA) and 1% penicillin-Streptomycin Solution at 37 °C in a humidified atmosphere containing 5% CO_2_. miR-18a-3p lentivirus inhibitor (miR-18a-3p-inhibitor) and negative control virus (NC) was ordered from Genechem Company (Shanghai, China). 20 μM miR-18a-3p-inhibitor or NC were transfected into the cells at ~50% density using HitransG P Transfection Reagent (Genechem, Shanghai) according to the manufacturer's protocol guide.

### BrdU staining

1×10^4^ cells were cultured in 24-well plates overnight at 37 °C, 5% CO_2_. DMEM medium with different concentration of HHT as added to each well, and cells were cultured at 37 °C for 2 days. Add 10 μg/ml BrdU (Abcam, USA,) and incubation for 2 h. The cells were then washed with PBS and fixed in 4% paraformaldehyde for 30 min. Subsequently, 2M HCl were used to treat the cells 30 min at room temperature. After being washed three times with PBS buffer, the cells were treated with 0.5% Triton X-100 and then blocked with 5% goat serum (ZSGB-Bio, Beijing, China) for 1 h. The cells were incubated with a monoclonal rat primary antibody against BrdU (1:1000) at 4 °C overnight,after being washed three times with PBS buffer and followed by incubation with Alexa FluorR® 488 goat anti-rat IgG secondary antibody (H+L; 1:250, Invitrogen). The nuclei were stained with DAPI (1:500). The percentage of BrdU staining was calculated from at least 10 microscopic fields.

### Cell proliferation and colony formation assays

The cell proliferation ability was assessed with CCK-8 and colony formation assay. For the CCK-8 assay, after treated with difference concentration of HHT or infected miR-18a-3p inhibitor or NC, the cells were cultured for 24 h, 48 h and 72 h separately. Then treated the cells with CCK-8 reagent (KeyGEN BioTECH, Jiangsu, China) and further cultured for 2 h according to the manufacturer's instructions. The optical density was measured using the spectrophotometer (Glomax Multi Detection Systerm, Promega, USA) at 450 nm. Each group of experiments included five replicates and repeated three times.

For the colony formation assay, 8×102 cells were cultured in 6-well plate. The cells were exchanged medium every three days. After 15 days, discarded medium, fixed the plates with 100% methanol for 15 min and then stained with crystal violet for 10 min. The number of colonies was observed and calculated. All experiments were performed in triplicate.

### Wound-healing and Transwell assay

The cells were seeded in 12-well plate with DMEM medium for 48 h. Then the cell monolayer was wounded by 200 μl pipette tip scraping. The exfoliated cells were washed with PBS and continue cultured for additional 24 h, 48 h, 72 h with different concentrations of HHT, transfected miR-18a-3p inhibitor or NC respectively. The speed of wound closure was observed and photographed using a microscope (Nikon, Tokyo, Japan). Cell mobility was assessed by measuring three randomly perpendicular wound width.

For the transwell assay, Corning Incorporated Transwell Chambers (Corning, 8 μm, NY, USA) were used to detect the migration capacity of the cells. Placed the chambers into the 24-well plate that contained culture medium supplemented with 20% FBS as a chemo-attractant, 2×10^4^ cells were suspended in serum-free medium and loaded into the upper chamber. Cells were incubated at 37 °C and allowed to invade through the membrane pores. After 15h, the non-invading cells were removed and the lower side of the membrane were fixed with 100% methanol and stained with crystal violet. The cells were photographed under a microscope and the cell migration was observed to calculate the number of transmembrane cells.

### Cell apoptosis detection

The cells were seeded in 6-well plates and treated with different concentration of HHT, transfected miR-18a-3p inhibitor or NC for 48 h. Cells were harvested and washed with cold PBS and then resuspended in 500 μL of binding buffer (BestBio, Shanghai, China). Incubate the cells with 5 μL Annexin V-FITC (BestBio, Shanghai, China) and 10 μL Propidium lodide (BestBio, Shanghai, China) at room temperature for 15 min in the dark. Samples were examined using BD flow cytometer and the data were analyzed with FlowJo software (TreeStar Corporation, Ashland, OR, USA).

### Western blotting

After treating with different concentration of HHT, tranfected miR-18a-3p inhibitor or NC, the cells were harvested in the RIPA lysis buffer (Beyotime Biotechnology, Shanghai, China). The total protein was extracted and the concentration of each sample was determined by BCA protein assay reagent kit (Thermo Fisher Scientific, Inc.). The supernatants containing total protein were mixed with a corresponding volume of 5×SDS loading buffer (Beyotime Biotechnology, Shanghai, China) and heated at 100 °C for 10 min. The lysates of 30 μg were electrophoresed on 10% SDS-PAGE and transferred to PVDF membranes. The proteins were blocked containing 5% defatted milk for 2 h at room temperature. Subsequently, primary antibodies were incubated at 4°C overnight. Followed by incubation with conjugated secondary antibodies (anti-rabbit, 1:10,000, cat: #7074, or anti-mouse, 1:10,000, cat: #7076, CST, Inc.) for 1.5 h at room temperature. To ensure equal amounts of sample protein applied for electrophoresis, GAPDH was used as an internal control.

### RNA isolation and qRT-PCR

Total RNA was extracted from patient tissue and plasma samples using the Trizol reagent (Invitrogen) and purified with miRNA Isolation Kit (Ambion, Austin, TX, USA) according to the protocol. The purity and concentration of RNA were determined from OD260/280 readings using spectrophotometer NanoDrop2000 (Thermo Fisher Scientific). RNA integrity was determined by 1% formaldehyde denaturing gel electrophoresis.

The cDNA was synthesized by the Superscript Reverse Transcription System (Invitrogen). RT-qPCR was performed using TB Green qPCR Mastermix (TaKaRa, Japan) on LightCycler® 480 real-time PCR Platform (Roche). The qRT-PCR reaction in a total volume of 20 μL system, including 0.8 μL/10 μM forward/reverse primers, 10 μL TB Green qPCR Mastermix, 2 μL cDNA, and 6.4 μL double-distilled water. The cycling program is 95 °C for 30 sec, followed by 40 cycles of 95 °C for 8 sec and a pre-selected annealing temperature for 30 sec. The primers (Table 1) were synthesized Sangon biotech (Shanghai) Co. Ltd. The relative expression of genes were calculated using the ΔΔCT method.

After treatment with HHT at 37 °C for 2 days, the cells were harvested and total RNA was isolated using Trizol reagent (Invitrogen, CA, USA) according to the manufacturers' instructions. The purity and concentration of RNA were determined by using spectrophotometer NanoDrop2000 (Thermo Fisher Scientific), cDNA synthesis was performed according to the reverse transcription kit (TaKaRa, Shanghai, China) manufacturer's protocols. The PCR amplification was performed with specific primers and carried out using the SYBR-Green PCR system (Takara Bio, Inc). GAPDH served as internal control. Calculation of the relative expression of each gene was quantified by the 2^-ΔΔCt^ method.

### microRNA sequencing and analysis

RNA samples were submitted to CapitalBio Technology (Beijing, China) for microRNA sequencing and analysis. The data read from high-throughput sequencing was first screened, then 18 to 30 nt clean reads are mapped to the Rfam database (http://rfam.janelia.org/) and the Repbase database (http://girinst.org/repbase/), short RNA maps to miRNA precursors of the human reference genome, and mature miRNAs are deposited into the miRBase 21.0 database (http://www.mirbase.org/). Using the existing mirdeep2 software to identify the minimum free energy of the Dicer lysate and the unannotated small RNA marker in the previous step by exploring the secondary structure, and predicting new candidate miRNAs for unannotated short reads not mapped to miRBase 21.0 miRNA expression levels were estimated by TPM (transcript per million). Differential expression analysis of two flowering phases was performed using the R package edge R/limma. miRNAs that had changed ratios of more than 2 or less than 0.5 (Fold change Log_2_ > 1 or < -1) and *P* < 0.05 was set as the threshold for significant differential expression by default. Target gene prediction of differential expression miRNAs was performed using the miRanda. A GO functional enrichment analysis and KEGG pathway enrichment analysis were performed for Target gene using the KOBAS 3.0 software (http://kobas.cbi.pku.edu.cn). GO terms and pathway terms with *P*-value less than 0.05 were considered significantly enriched by target genes.

Differentially expressed genes were validated by quantitative real-time PCR (qPCR). The primer sequences for the miR-18a-3p and U6 were listed in supplements Table 1.The data were normalized using the U6 transcriptsfor miRNA. The relative expression of each gene was quantified by the 2-ΔΔCt method.

### *In vivo* tumorigenicity assay

5-6 week old female BALB/c male nude mice (bought from Animal Center of the Chinese Academy of Science, Shanghai, China) were housed and maintained under specific pathogen-free conditions following animal ethic guideline of the Animal Care and Use Committee of Ningxia Medical University. 2×10^7^ MCF-7 cells were injected subcutaneously into each mouse. Treatment was initiated when tumors reached a volume of 50-150 mm^3^. Tumor volume was calculated as the largest length × width was 2 × 0.5 and tumor diameter was measured three times every week. Then divided the mice into control group and HHT treatment group, each group contained seven animals. HHT was diluted in PBS and was intraperitoneally injected with a daily dose of 50 μg/kg for 10 days. The animals were sacrificed and tumor mass was collected for further study.

### Statistical analysis

Statistical analyses were performed using SPSS23.0 software (IBM, USA) and GraphPad Prism 7.0 (GraphPad Software, USA). All assays were conducted three times and data were presented as mean ± SD. Statistical differences were determined by* t*-test. The results were considered significant when *P* values< 0.05.

## Results

### HHT inhibited breast cancer cells growth

**Figure 1:**
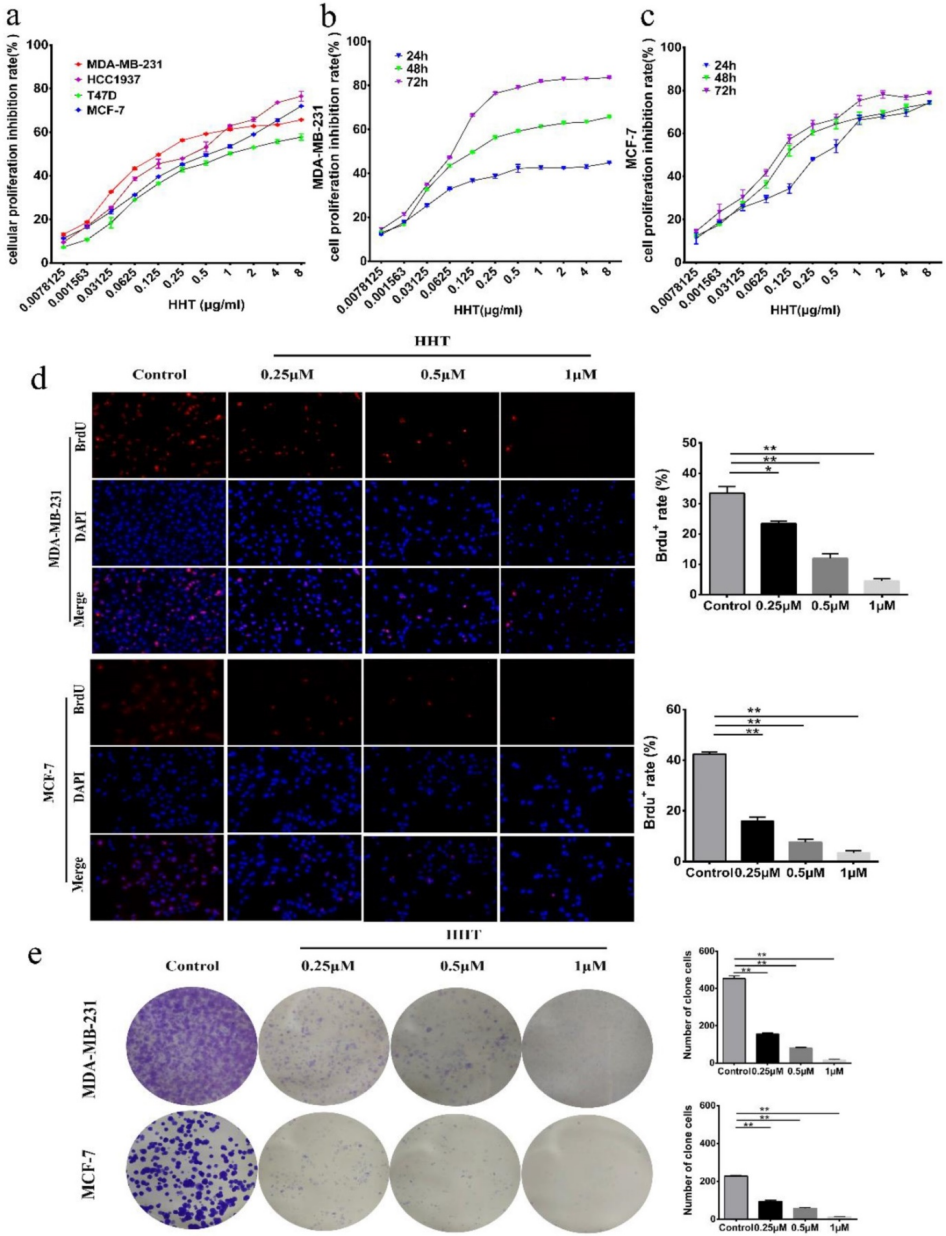
** HHT inhibited breast cancer cells growth and proliferation. a.** Effects of different concentrations of HHT on breast cancer cells, including MDA-MB-231, MCF-7,T47D and HCC1937 cells, while screening drug-sensitive cell lines. **b.** MDA-MB-231 cell was treated with different concentrations of HHT for 24 h, 48 h and 72 h. **c.** MCF-7 cell was treated with different concentrations of HHT for 24 h, 48 h and 72 h. Cellular proliferation inhibition rate was evaluated using CCK-8 assays, and the data are presented as the means ± SD. **d.** Images of MDA-MB-231 and MCF-7 cells positive for BrdU staining after treatment with indicated concentration of HHT, Scale bar =100 µm. The histogram demonstrates the results of BrdU-positive rate in MDA-MB-231 and MCF-7 cells. **e.** Colony formation assays were used to investigate the colony formation abilities of MDA-MB-231 and MCF-7 cells after treatment with the indicated concentrations of HHT. All data were analysed using unpaired Student's t-tests and are shown as the means ± SD. *p< 0.05, **p< 0.01.

To investigate the effect of HHT on breast cancer cell growth, 4 breast cancer cell lines (MDA-MB-231, HCC-1937 MCF-7 and T47D) were used to examine the effects. After treated with a series of concentrations of HHT for 48 h, the inhibition rates of proliferation of all tested cells were significantly increased in a dose-dependent manners (Fig. 1a), demonstrating that HHT has a broad anti-cancer effects on human breast cancer cells. In addition, the IC50 of HHT for each breast cancer cell line showed that MDA-MB-231 and MCF-7 cells were more sensitive to HHT compared to other cell lines (Table 2). Therefore, we selected these two cells for subsequent experiments. CCK-8 assays revealed that HHT significantly inhibited MDA-MB-231 and MCF-7 cell growth in a dose-dependent manner and time-dependent manner (Fig. 1b, c). BrdU staining assay showed that HHT significantly decreased the percentage of BrdU positive cells on MDA-MB-231 and MCF-7 cells in a dose-dependent manners (Fig. 1d). Colony formation assays were performed to further investigate the effect of HHT on the self-renewal capability of the cells. The results showed that the colony numbers were smaller and fewer when treated with HHT (Fig. 1e). These results demonstrated that HHT remarkably inhibited breast cancer cells growth.

### HHT inhibited migration and invasion of breast cancer cells

**Figure 2:**
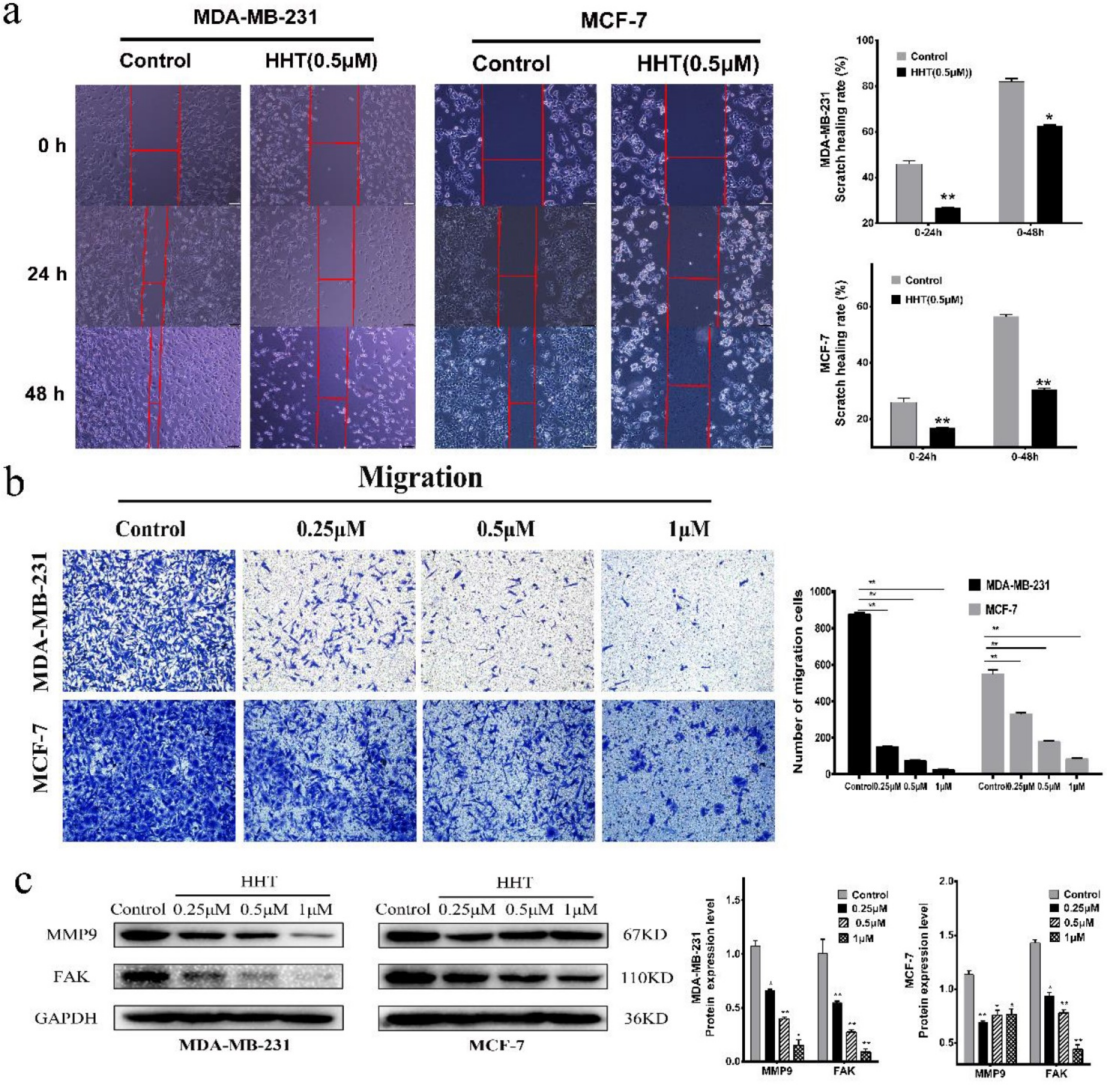
** HHT inhibited migration and invasion abilities of breast cancer cells. a.** *In vitro* migration ability of MDA-MB-231 and MCF-7 cells were detected using scratch wound healing assay after different concentrations of HHT for 48 h. **b.** Transwell migration assays of MDA-MB-231 and MCF-7 cells treated with HHT. **c.** The expression of MMP9 and FAK were analysed by Western blot. GAPDH was used as the control. All data were analysed using unpaired t-tests and are shown as the means ± SD. *p< 0.05, *p< 0.01.

The effects of HHT on the migration and invasion abilities of breast cancer cells were also evaluated via transwell and wound-healing assay. The results showed that the cell scratch healing rate was significantly lower than that of the control group when treated with HHT. This experiment was consistent with the transwell experiment results, indicating that HHT can inhibit the migration ability of breast cancer cells. The statistical results are showed as representative histograms (Fig. 2a).

Compared with the control cells, the cell invasion ability was significantly inhibited when treated with HHT. The number of invasion cells of MDA-MB-231 and MCF-7 was obviously decreased. The invasion inhibition of HHT on the cells was in a dose-dependent manner (Fig. 2b). We further evaluated the expression of FAK and MMP9 by Western blot assays. The results showed that the expression of two proteins was deregulated after treatment with HHT (Fig. 2c). The results suggested that HHT inhibited migration and invasion abilities of breast cancer cells.

### HHT induced breast cancer cells apoptosis

**Figure 3:**
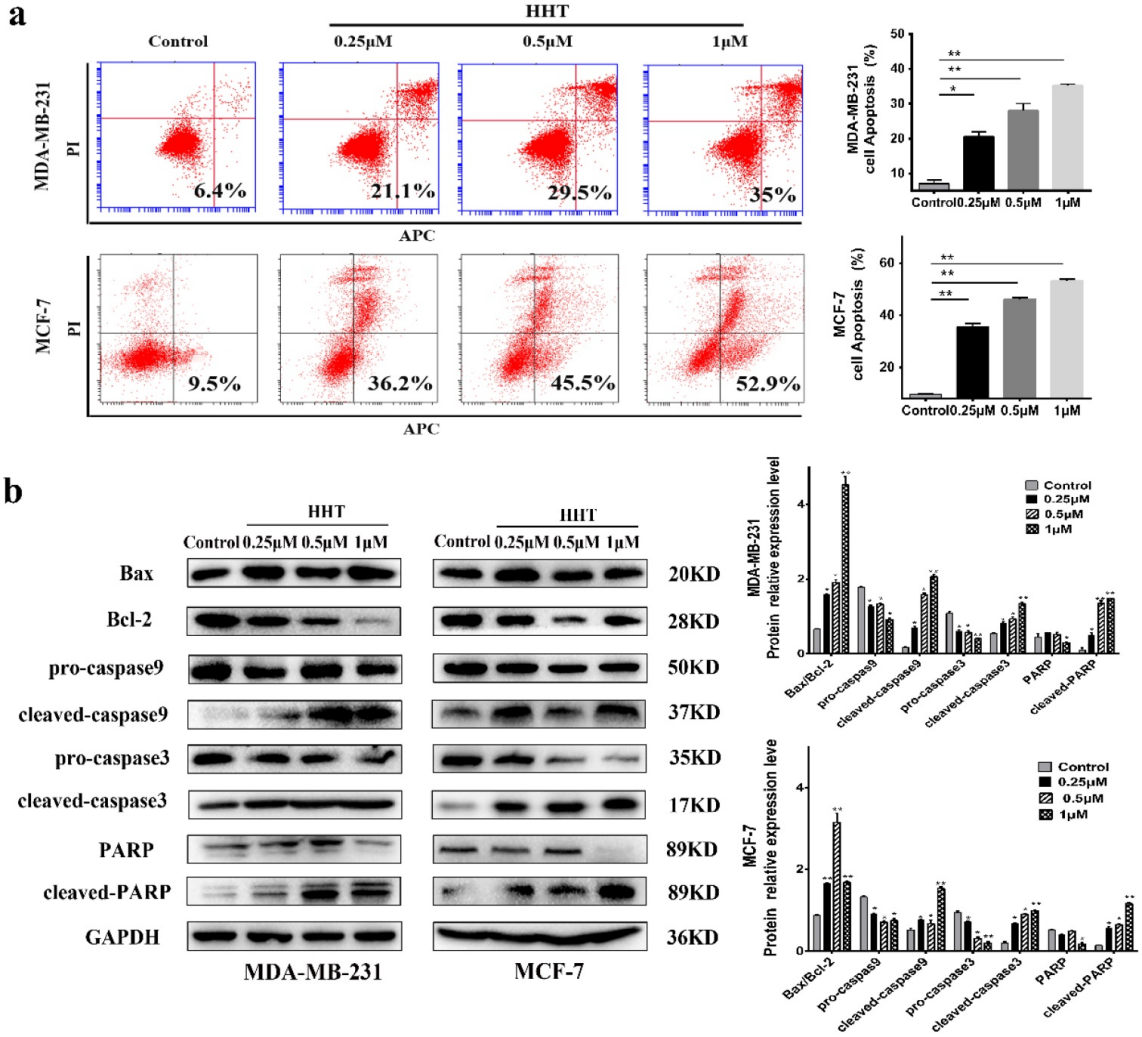
** HHT induce breast cancer cells apoptosis. a.** MDA-MB-231 and MCF-7 cells were treated with HHT for 48h, which caused a significant increase the proportion of apoptosis cells in a dose-dependent manner via flow cytometry. **b.** Western blot analysis of apoptosis regulatory-proteins after different concentrations of HHT for 48 h. GAPDH was used as the control. All data were analysed using unpaired t-tests and are shown as the means ± SD. *p< 0.05, **p< 0.01.

With AnnexinV/ PI staining and flow cytometry analysis, we evaluated the effects of HHT on the cells apoptosis. The results demonstrated that the apoptosis of MCF-7 and MDA-MB-231 cells being induced by HHT in a dose-dependent manner (Fig. 3a). The Western bloting showed that the expression of Bax, cleaved-caspase 3, cleaved-caspase 9, and cleaved PARP protein were increased when treated with HHT, while Bcl2, pro-caspase 3, pro-caspase 9, and PARP protein expression were decreased (Fig. 3b).

### HHT suppressed miR-18a-3p expression in breast cancer cells

**Figure 4:**
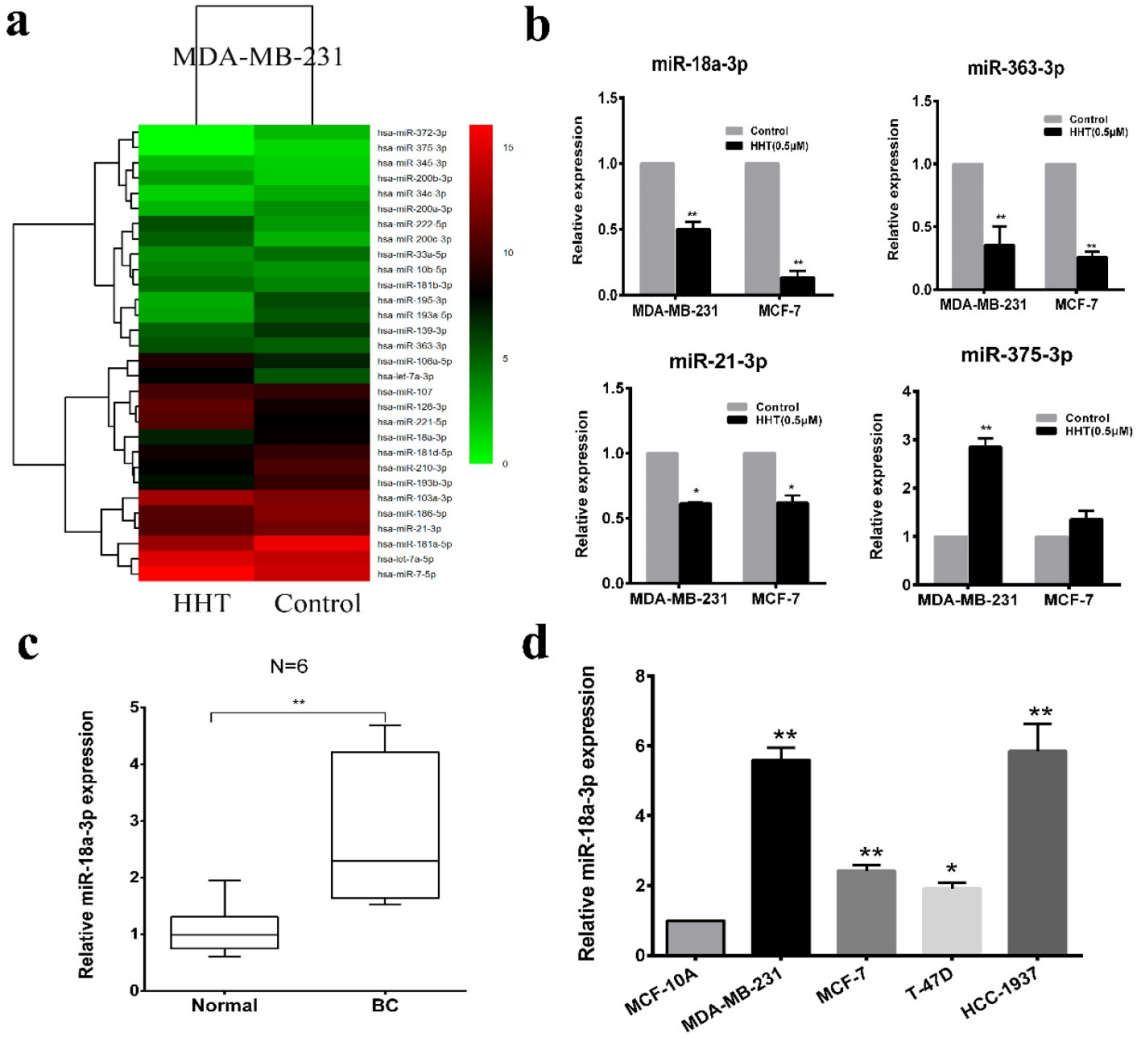
** HHT suppressed miR-18a-3p expression in breast cancer cells. a.** The heatmap shows the 30 significantly increased and decreased miRNAs in MDA-MB-231 after HHT treatment by small RNA sequencing. **b.** Relative expression of the 4 indicated miRNAs listed in (a) measured by RT-qPCR in MDA-MB-231 and MCF-7 cells. **c.** Relative expression of miR-18a-3p from 6 breast cancer tissues and adjacent non-tumor tissues. The expression levels of miR-18a-3p in 6 breast cancer cell lines and one normal breast epithelial cell line, MCF-10A.

To investigate the mechanism of HHT induced breast cancer cell apoptosis. High-throughput sequencing was used for microRNA sequencing in the cells with HHT treatment. A total of 379 miRNA candidate was selected based on the screening criteria as a fold-change > 1.5 or ≤ 1.5 and a *P*-value < 0.05. We then narrowed the analysis scope and verified 30 aberrantly expressed miRNAs, including 15 upregulated and 15 downregulated (Fig. 4a). Quantitative real-time PCR validated that miR-18a-3p, miR-21-3p and miR-363-3p were significantly low-expressed in the HHT treatment group, while miR-375-3p expression increased (Fig. 4b). Considering miR-18a-3p was high expression in 6 paired breast cancer tissues and the cell lines before HHT treatment (Fig. 4c, d). We proposed that miR-18a-3p might be the cause of HHT-inhibited breast cancer growth.

### Downregulating miR-18a-3p enhanced HHT functional in breast cancer cells

**Figure 5:**
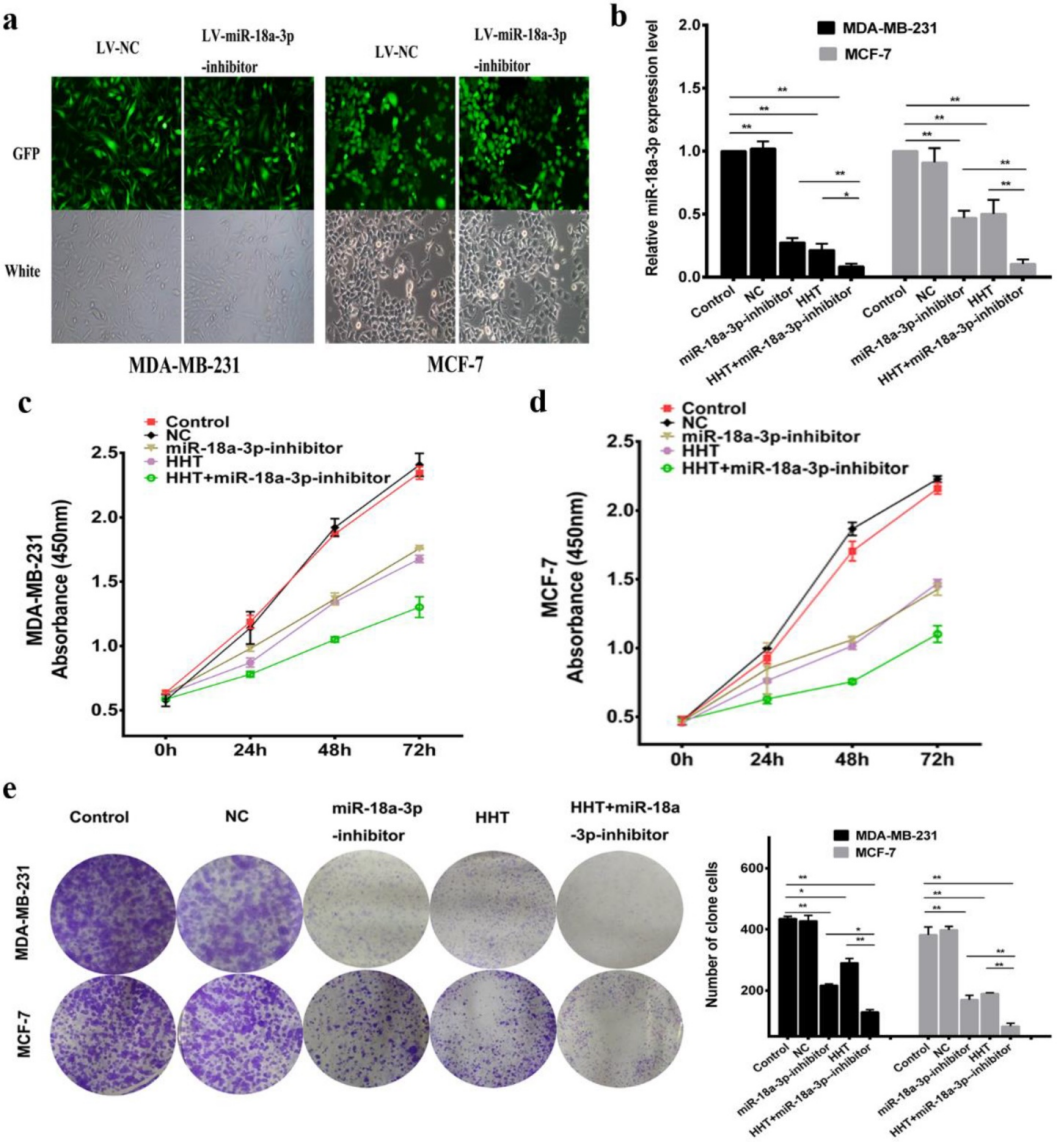
** Downregulating miR-18a-3p increased HHT-induced inhibition of cell growth. a.** Observed green fluorescence under the fluorescence microscope and verified the infection efficiency of lentivirus in both breast cancer cells. **b.** Validated the expression of miR-18a-3p when infected with LV- miR-18a-3p or LV-NC by qRT-PCR.The cell proliferative ability was detected by MTT (c and d) and clone formation assays (e). All data were analysed using unpaired t-tests and are shown as the means ± SD. *p< 0.05, **p< 0.01.

**Figure 6:**
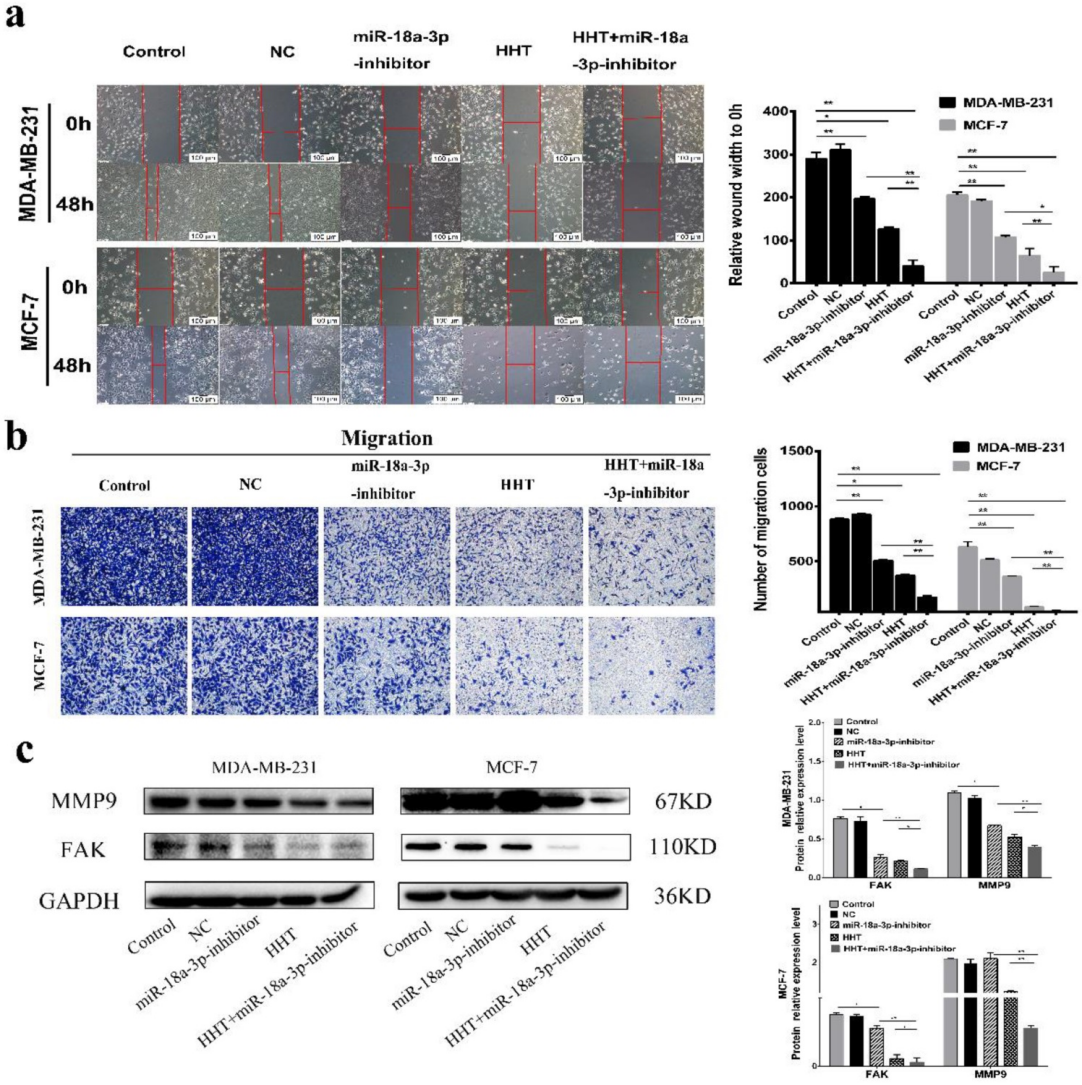
** Downregulating miR-18a-3p increased HHT-induced inhibition of cell migration. a.** Migration ability were detected using scratch wound healing assay (scale bar = 100 µm). **b.** Cell metastatic was assessed using transwell assay. **c.** The expression of MMP9 and FAKwere analysed by Western blot. GAPDH was used as the control. *P< 0.05, **P< 0.01.

Based on previous hypothesis, the miR-18a-3p inhibit lentivirus vector (miR-18a-3p inhibitor) being constructed and transfected into the breast cancer cell lines (Fig. 5a). miR-18a-3p expression was successfully decreased in MDA-MB-231 and MCF-7 cells treated by miR-18a-3p inhibitor. Furthemore, miR-18a-3p inhibitor, the expression of miR-18a-3p in the cells were significantly decreased (Fig. 5b). CCK-8 and clone formation assays verified the same conclusion (Fig. 5c, d, e). Wound healing and transwell assays were also performed to detect the effect of miR-18a-3p on breast cancer cells. The results revealed that inhibition of miR-18a-3p enhances the effect of HHT on breast cancer cells invasion and metastasis (Fig. 6a, b). Western blot proved that the metastasis associated proteins MMP9 and FAK expression were inhibited in the miR-18a-3p inhibitor group, and inhibited expression of miR-18a-3p can enhance the function of HHT on breast cancer cells (Fig. 6c). The results were consistent with cell phenotypic experiments. Taken together, these data proved that miR-18a-3p plays an important role in regulating the biological characteristics of breast cancer cells with HHT treatment.

**Figure 7:**
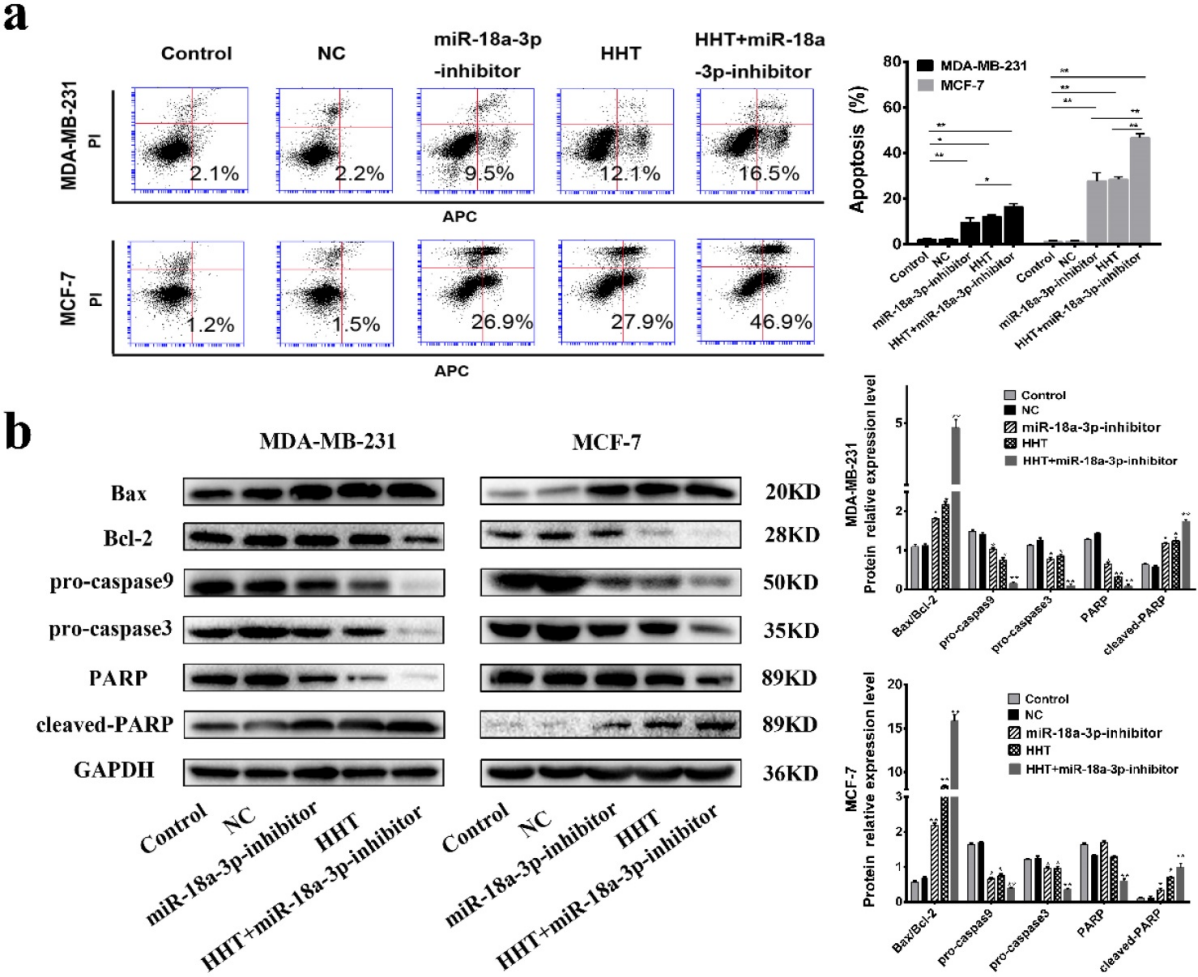
** Downregulating miR-18a-3p increased HHT-induced cell apoptosis. a.** Flow cytometry was used to detect the effect of miR-18a-3p-inhibitor on the apoptosis rate of two breast cancer cells. **b.** Western blot analysis of the effect of miR-18a-3p-inhibitor on the expression of apoptosis-regulated proteins in breast cancer cells.GAPDH was used as the control. All data were analysed using unpaired t-tests and are shown as the means ± SD. *p< 0.05, **p< 0.01.

Flow cytometry indicated that the proportion of apoptotic cells in the miR-18a-3p inhibitor group was markedly increased. Downregulation of miR-18a-3p expression enhances HHT-induced apoptosis in breast cancer cells Simultaneously (Fig. 7a). Western blot showed that the expression of Bcl2, pro-caspase3, pro-caspase 9 and PARP were all decreased, while Bax, cleaved-caspase 3, cleaved-caspase 9, and cleaved PARP expression were increased. The results indicated that downregulation of miR-18a-3p expression enhanced HHT-induced apoptosis in breast cancer cells (Fig. 7b).

### HHT inhibited breast cancer cells growth via miR-18a-3p-AKT-mTOR signaling pathway

**Figure 8:**
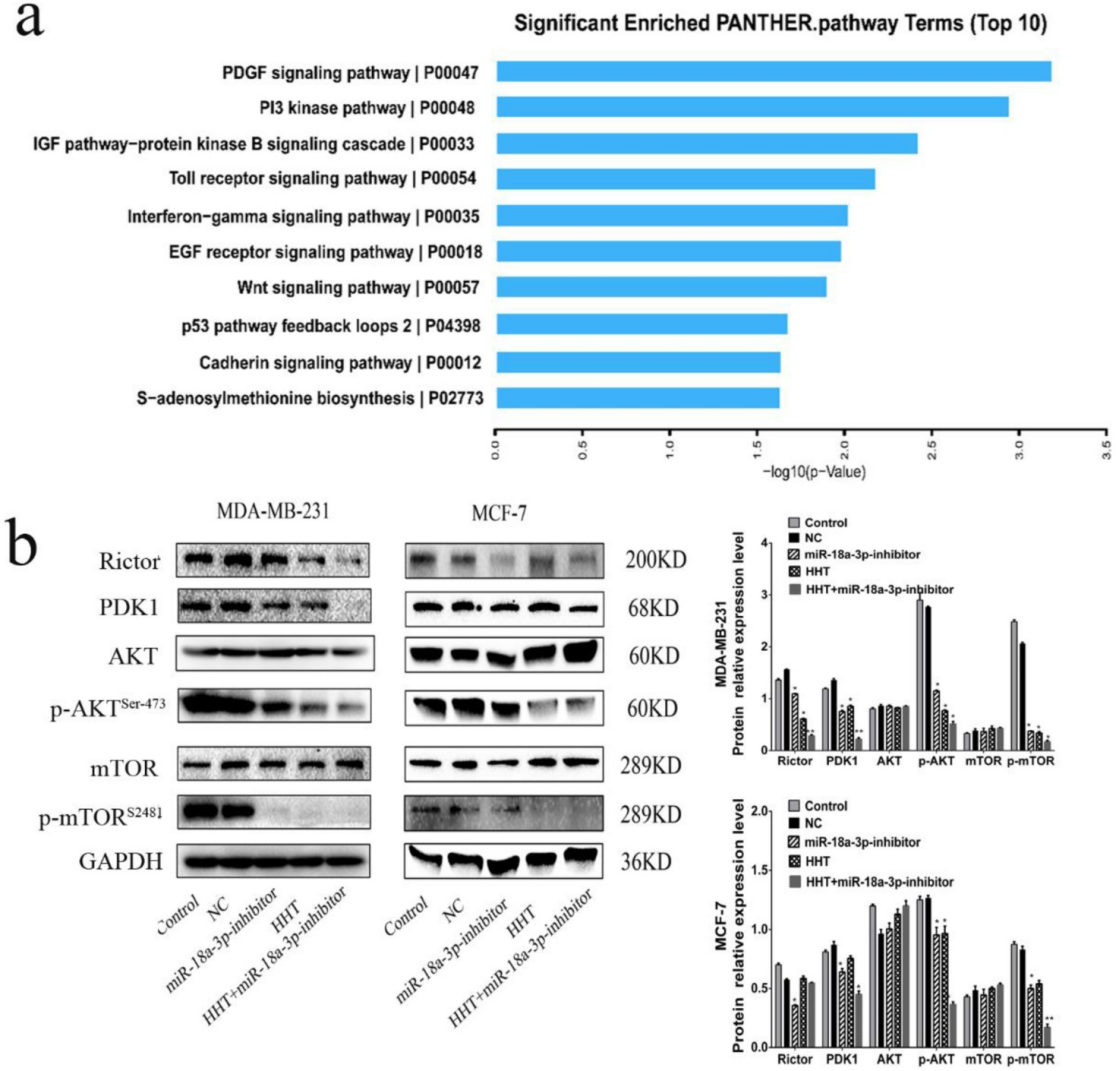
** HHT inhibits the expression of genes related to AKT/mTOR signaling pathway. a.** KEGG pathway enrichment analysis of genes downregulated (determined via whole transcriptome analyses (RNA-seq)) in both cells after treament with HHT. The top 10 signaling pathway based on fold enrichment are shown. **b.** Western blot analyses of Pathway related protein, including AKT, p-AKT, PDK1, mTOR and p-mTOR. GAPDH was used as the control.

The AKT-mTOR signaling pathway has been reported to regulate numerous cellular processes, such as cell growth, proliferation and migration 13, 14. When analysis the relationship between the AKT-mTOR signaling pathway and miR-18a-3p, we found that AKT-mTOR signaling pathway were associated with miR-18a-3p expression (Fig. 8a). Western blot was conducted to identify the relationship and its influence for HHT treatment. The results showed that the expression of Rictor, PDK1, p-mTOR (ser-2481) and p-AKT (ser-473) were all decreased when treated the cell with HHT or miR-18a-3p inhibitor. When combined treated the cells with HHT and miR-18a-3p inhibitor, the inhibition of AKT-mTOR signaling pathway can be enhanced (Fig. 8b). The results suggested there have a miR-18a-3p-AKT-mTOR signaling pathway axis in BC cells. HHT suppressed breast cancer cell proliferation, migration and induced apoptosis through miR-18a-3p-AKT-mTOR signaling pathway.

### HHT inhibits tumor growth *in vivo*

**Figure 9:**
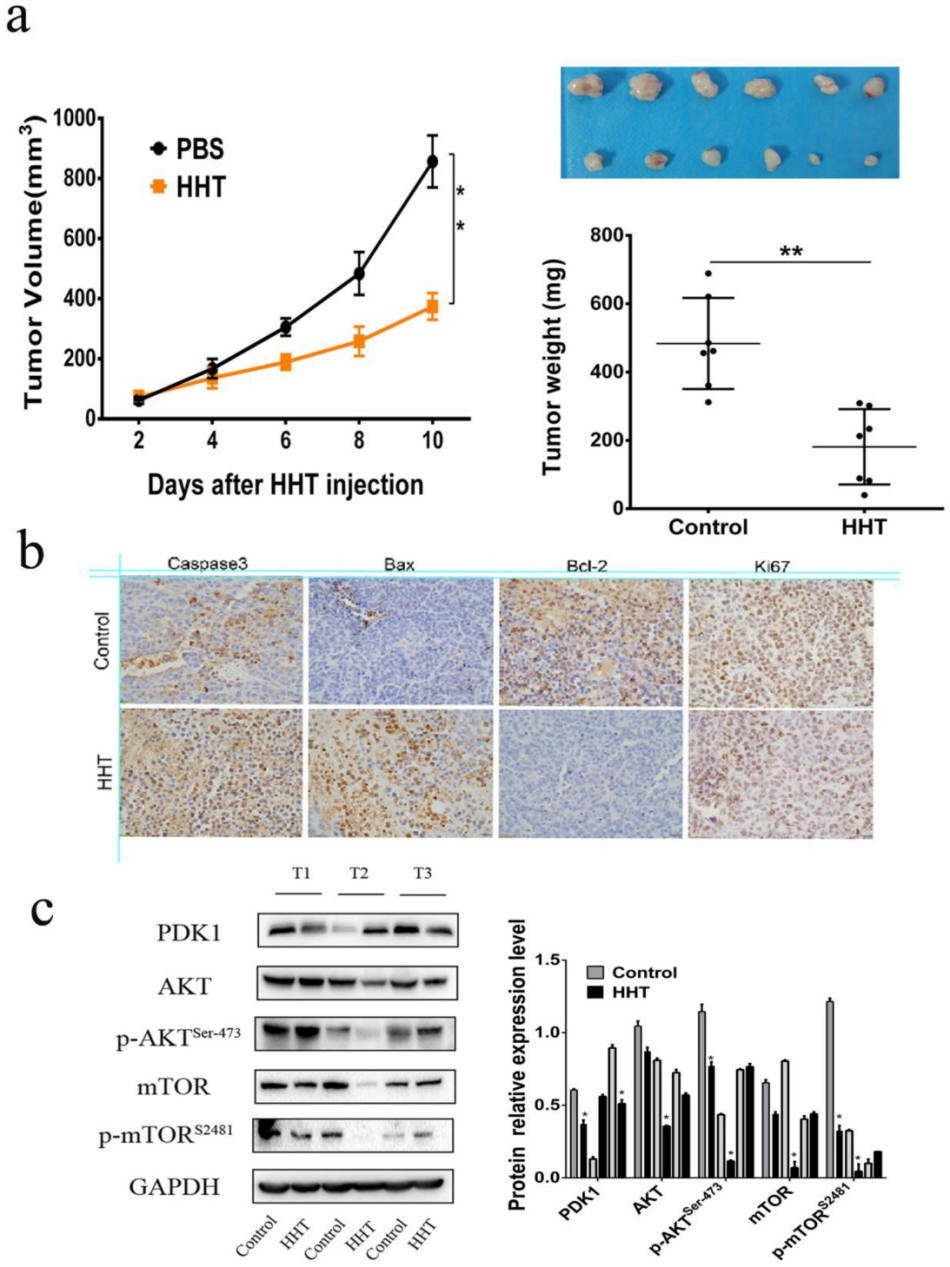
** HHT treatment led to tumor growth slowed *in vivo*. a,c.** Tumor diameters were measured after therapy 3 times every week, and tumor volumes were calculated. Values are mean ± SD. *p < 0.05. b. MCF-7 cells were injected subcutaneously into BALB/c male nude mice. After cycle of therapy, animals were sacrificed and tumors were excised.

To further validate the effects of HHT *in vivo*, MCF-7 cells were injected subcutaneously into the BALB/c nude mouse. 10 days after injection, the mouse were divided into two groups and treated with PBS and 50 μg/kg HHT separately. After HHT treatment, the xenografts volume and weight were significantly lower than those in the PBS group (Fig. 9a-c). The findings suggested that HHT has functional of inhibited tumor growth *in vivo*. Immunochemistry staining results proved that caspase-3, Bax expression were increased in the tissues treated by HHT. Meanwhile, Bcl-2 and Ki-67 expression were decreased in the group. Western blotting being used to further illucide the influence of HHT on the AKT-mTOR signaling pathway in xenografts. The results showed p-mTOR (ser-2481) and p-AKT (ser-473) were all decreased when treated with HHT, which were consistent with those of the cell treatment group.

## Discussion

Breast cancer was one of the most common malignancy among women all over the world 1. Despite significant advances therapeutic have been made, the occurrence of metastasis still leads to poor prognosis and low survival for breast cancer. Therefore, to find new medicine for breast cancer therapy has been gradually put on the agenda. Using natural compounds as anti-cancer medicines was always been applied in different cancer. Homoharringtonine (HHT), an alkaloid which extracted from cephalotaxus, have been used for hematological malignancies treatment many years.

Recent reports have shown that HHT can be used for solid tumor treatment, especially in breast cancer. Mohamad Yakhni et al. reported that HHT can inhibit tumor growth of four different types of breast cancer cells *in vitro* 15. Isoharringtonine (IHT), a natural analogue of HHT, was found to have functional of decreasing the proliferation, migration, and breast cancer stem cell proportion via inhibition of the STAT3/Nanog pathway 16. It was also reported that HHT was sensitive to the triple-negative breast cancer (TNBC) cell lines, indicating that HHT was an effective medicine for triple-negative breast cancer 17. However, the underlying regulation mechanisms of how HHT treated breast cancer are not being fully explained. In this study, we demonstrated that HHT inhibited human breast cancer cell growth and proliferation *in vivo* and *in vitro*. Besides, HHT can also induce breast cancer cell apoptosis by regulated the expression of apoptosis-related protein like Bax/Bcl-2, Caspase3/Caspase9 and PARP.

microRNA as one a noncoding RNA, had involved in different biological processes, including cell growth, proliferation, differentiation, metastasis and drug-resistance 18, 19.

An increasing number of evidences indicated that miRNAs play a crucial role in cancer proliferation and metastasis 20, 21. To further illustrate the molecular mechanism of how HHT inhibited BC proliferation, miRNA sequencing being used to analysis the PCR, 379 differentially expressed candidate miRNAs being identified. Among them, miR-18a-3p was significantly down-regulated in breast cancer cells after HHT treatment. Previous research has showed that miR-18a-3p has suppressed Dicer expression and increased Paclitaxel (PTX) resistance in TNBC cells 22. Our research suggested that HHT could inhibit the expression of miR-18a-3p. When treated the BC cell combined with HHT and miR-18a-3p inhibitor, the cell proliferation, differentiation were all being suppressed, while apoptosis of the cells being promoted. The results suggested that the functions of HHT on BC cells can be enhanced by down-regulation of miR-18a-3p.

**Figure 10:**
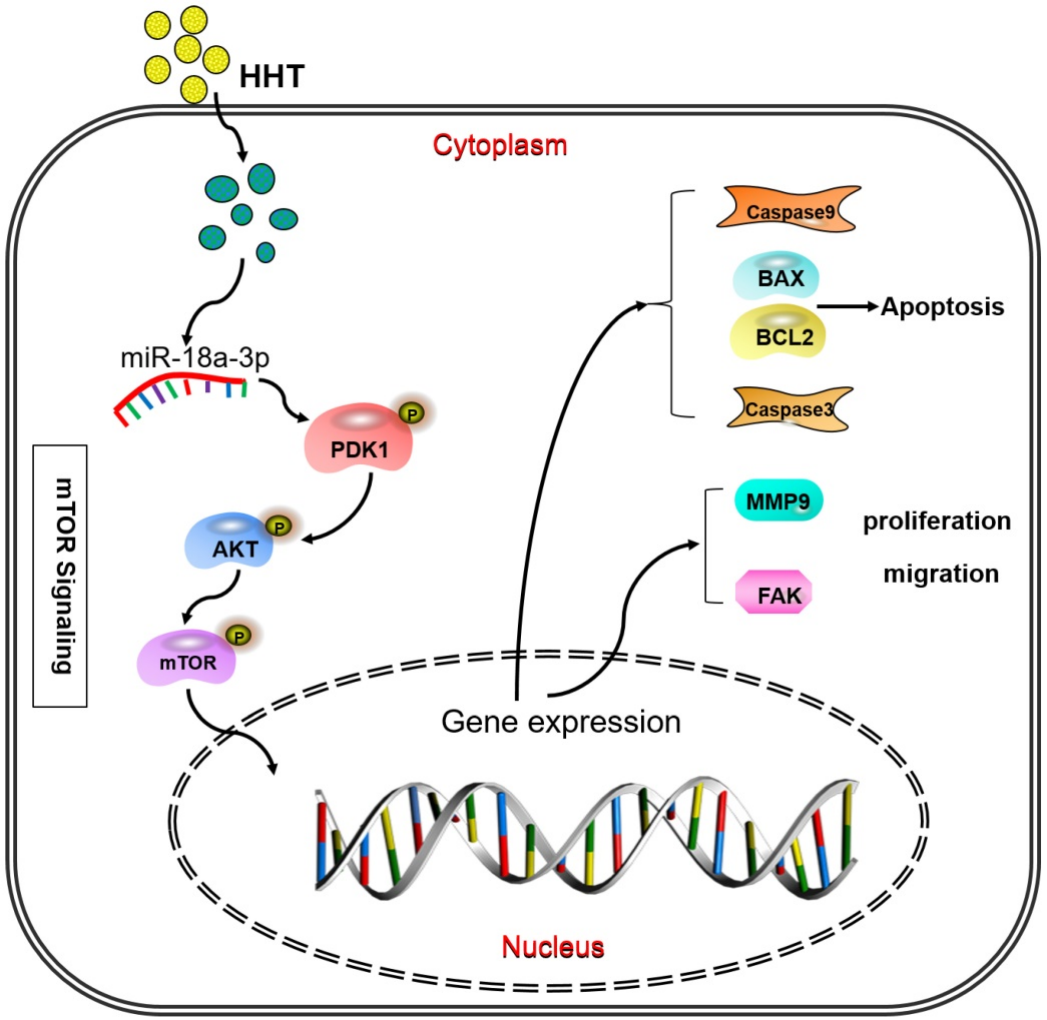
** Pattern diagram HHT influence miR-18a-3p and AKT/mTOR signaling pathway.** The pattern diagram demonstrated how HHT influence miR-18a-3p and AKT/mTOR signaling pathway expression and exhibited its function.

When analysis the signaling pathway related to the miR-18a-3p, we found that AKT/mTOR signaling pathway were significantly associated with miR-18a-3p. As we all know, mTOR is a key kinase which was activated by phosphoralytion of AKT. The AKT-mTOR signaling pathway could regulate cancer cell proliferation, growth, survival and angiogenesis 23, 24. It was reported that AKT-mTOR signaling pathway was involved in the process of HHT therapy for hematological malignancy 25, 26. Our previous research has confimed that HHT inhibited human colorectal cancer cell proliferation and induced the cell apoptosis by blockage of mTOR signaling pathway 27. In this study, we detected the proteins expression of mTOR signaling pathway after treated with miR-18a-3p inhibitor and HHT or both *in vitro* and *in vivo*. Bioinformatics analysis showed that there is a strong correlation between miR-18a-3p and mTOR signaling pathway. miR-18a-3p exercised its regulatory role through the AKT-mTOR signaling pathway. When treated the cell with miR-18a-3p inhibitor and HHT, the mTOR singnaling pathway expression being suppressed. The mouse xenografts experiment consisitant with the results. All the experiments proved that HHT could induce BC cell apoptosis and inhibit its proliferation by inhibition of miR-18a-3p and mTOR signaling pathway (Fig. 10).

In conclusion, our study proved that HHT suppressed breast cancer cells proliferation, migration and induced apoptosis through miR-18a-3p/AKT/mTOR signaling pathway. We therefore confirmed that HHT exhibits a strong anti-cancer activity in breast cancer cells. HHT may have the potential to be a promising medicine for the treatment of breast cancer.

## Figures and Tables

**Table 1:** The prime sequence for RT-qPCR

Gene Name	Primer Sequence (5'→3')		miRBase Accession number
hsa-miR-18a-3p	F	CGACTGCCCTAAGTGCTCC	MIMAT0002891
	R	AGTGCAGGGTCCGAGGTATT	
hsa-miR-363-3p	F	GCGAATTGCACGGTATCCA	MIMAT0000707
	R	AGTGCAGGGTCCGAGGTATT	
hsa-miR-21-3p	F	GCGCAACACCAGTCGATG	MIMAT0004494
	R	AGTGCAGGGTCCGAGGTATT	
hsa-miR-375-3p	F	CGGGTTTGTTCGTTCGGCT	MIMAT0000728
	R	GTGCAGGGTCCGAGGTATT	

The prime sequence designed for RT-qPCR, the primer were synthesized by Sangon biotech (Shanghai) Co. Ltd, China. Annotation: F for Up prime; R for Down prime.

**Table 2:** The IC50 of HHT on different breast cancer cells

The cells	IC50 (μg/ml)
MDA-MD-231	0.313
HCC1937	0.324
MCF-7	0.459
T47D	1.270

The inhibitory concentration rate (IC50) of HHT on different breast cancer cells was detected by CCK-8 assay. The results showed MDA-MB-231 and MCF-7 cells were more sensitive to HHT compared to other cell lines.
